# Multi-task Deep Learning of Myocardial Blood Flow and Cardiovascular Risk Traits from PET Myocardial Perfusion Imaging

**DOI:** 10.1007/s12350-022-02920-x

**Published:** 2022-03-10

**Authors:** Ming Wai Yeung, Jan Walter Benjamins, Remco J. J. Knol, Friso M. van der Zant, Folkert W. Asselbergs, Pim van der Harst, Luis Eduardo Juarez-Orozco

**Affiliations:** 1 Department of Cardiology, University Medical Center Groningen, University of Groningen, 9700 RB Groningen, The Netherlands; 2 Department of Cardiology, Division of Heart & Lungs, University Medical Center Utrecht, University of Utrecht, Heidelberglaan 1, Box 30.001, 9700RB Utrecht, The Netherlands; 3 Department of Nuclear Medicine, Cardiac Imaging Division Alkmaar, Northwest Clinics, Alkmaar, The Netherlands

**Keywords:** Deep learning, explainAI, Cardiovascular risk factors, Myocardial perfusion, flow reserve, Nuclear medicine, PET imaging, Medical image analysis

## Abstract

**Background:**

Advanced cardiac imaging with positron emission tomography (PET) is a powerful tool for the evaluation of known or suspected cardiovascular disease. Deep learning (DL) offers the possibility to abstract highly complex patterns to optimize classification and prediction tasks.

**Methods and Results:**

We utilized DL models with a multi-task learning approach to identify an impaired myocardial flow reserve (MFR <2.0 ml/g/min) as well as to classify cardiovascular risk traits (factors), namely sex, diabetes, arterial hypertension, dyslipidemia and smoking at the individual-patient level from PET myocardial perfusion polar maps using transfer learning. Performance was assessed on a hold-out test set through the area under receiver operating curve (AUC). DL achieved the highest AUC of 0.94 [0.87-0.98] in classifying an impaired MFR in reserve perfusion polar maps. Fine-tuned DL for the classification of cardiovascular risk factors yielded the highest performance in the identification of sex from stress polar maps (AUC = 0.81 [0.73, 0.88]). Identification of smoking achieved an AUC = 0.71 [0.58, 0.85] from the analysis of rest polar maps. The identification of dyslipidemia and arterial hypertension showed poor performance and was not statistically significant.

**Conclusion:**

Multi-task DL for the evaluation of quantitative PET myocardial perfusion polar maps is able to identify an impaired MFR as well as cardiovascular risk traits such as sex, smoking and possibly diabetes at the individual-patient level.

**Supplementary Information:**

The online version contains supplementary material available at 10.1007/s12350-022-02920-x.

## Introduction

Advanced medical imaging has boosted our capacity to diagnose both subclinical and clinical cardiovascular pathology without the constant need for invasive procedures. It has improved disease characterization and has proven helpful for prognostic evaluation. In the last decades, state-of-the-art imaging has increased its temporal and spatial resolution at a pace influenced by that of computational development (Moore’s law) offering a stream of data of which processing and interpretation may overwhelm the analytical workflows of both researchers and clinicians.^1^

Yet, it is suspected that the information contained in the images resulting from techniques such as coronary computed tomography angiography and positron emission tomography (PET) may not be fully harnessed through conventional analyses, which currently translates image attributes into simple and univariate proxies (e.g. calcium score for the former and summed stress score for the latter). Such biomarkers, albeit pragmatic and certainly interpretable, may omit a substantial proportion of the information contained in the images. As such, developments in imaging quality may have only marginally enhanced our understanding of the dynamics of cardiovascular disease.

Deep learning (DL) corresponds to a series of machine learning algorithms based on (convolutional) neural networks and has revolutionized image recognition in various fields of knowledge. DL can boost performance in image analysis through artificial learning of complex high-dimensional patterns in large datasets,^2^ which then are used to optimize classification tasks. DL has already delivered exciting breakthrough proofs of concept when applied in several pathological conditions including coronary artery disease as studied through SPECT (CAD).^3–7^ Furthermore, it has been suggested that DL analysis of standardized medical imaging, such as retinal images, may allow the characterization of chronic diseases that signify added cardiovascular risk through comorbidity.^8^

Presently, studies on the implementation of DL for the identification of myocardial ischemia in PET imaging are lacking. And it is unknown whether DL analysis of myocardial perfusion images may provide insights into patterns associated with the presence of cardiovascular risk traits. Hence, the present report evaluated the performance of DL in the identification of an impaired myocardial flow reserve (MFR) and cardiovascular risk traits to explore complex DL-derived patterns associated with such factors in quantitative PET myocardial perfusion imaging polar maps at the individual patient-level.

## Materials and Methods

### Study Population

From the population referred to quantitative PET myocardial perfusion imaging due to suspected myocardial ischemia between 2015 and 2017 at the department of nuclear medicine of the Northwest Clinics, Alkmaar, The Netherlands, the data of 1,185 patients was retrospectively collected and included in the present analysis. Patients with prior myocardial infarction (MI) or revascularization (either through PCI or CABG) were excluded from the present study.

All patients provided written informed consent for the use of their anonymous data for scientific purposes. In addition to the standard imaging protocol and clinical management, no measurements or actions affecting the patient were performed. The study was approved by the institutional research department and performed in accordance with the Declaration of Helsinki. The approval of the local ethical committee for the present study was not necessary since the study does not fall within the scope of the Dutch Medical Research Involving Human Subjects Act (section 1.b WMO, 26th February 1998).

### Clinical Data

Demographic (sex and age) and cardiovascular risk traits (hypertension, dyslipidemia, smoking and type 2 diabetes mellitus) were extracted from the electronic file system.

### PET Data Acquisition and Quantitative Perfusion Analysis

Every patient underwent a two-phase, namely rest and adenosine stress, PET scan with the use of ^13^N-ammonia as the perfusion radiotracer which was produced by the Cyclotron Noordwest BV. All image data were acquired in list mode on a Siemens Biograph-16 TruePoint TrueV PET/CT (Siemens Healthcare, Knoxville, USA) with the axial field of view of 21.6 cm. This 3D system consists of a 16-slice CT and a PET scanner with four rings of lutetium oxyorthosilicate (LSO) detectors. Patients were instructed to fast overnight and to avoid the consumption of methylxantines, caffeine-containing beverages or medications for 24 hours before the study. The details of the acquisition-reconstruction protocol have been published previously in detail.^9^

Based on the dynamic subsets, left ventricular contours were assigned automatically using the *Syngo MBF* software (Siemens Medical Solutions, Berlin, Germany) with minimum observer intervention when appropriate. With a previously described 2-compartment kinetic model for the aforementioned tracer, value of stress MBF, rest MBF and myocardial flow reserve (MFR) were computed and color-coded with a standard scale for each sample on the polar map through the resulting time-activity curves for quantification.^10^ An impaired MFR was defined as <2.0 in at least one of the 17 segments from the American Heart Association / American College of Cardiology standardized myocardial segmentation model.

### Image Analysis

#### Data flow and processing

**Figure 1:**
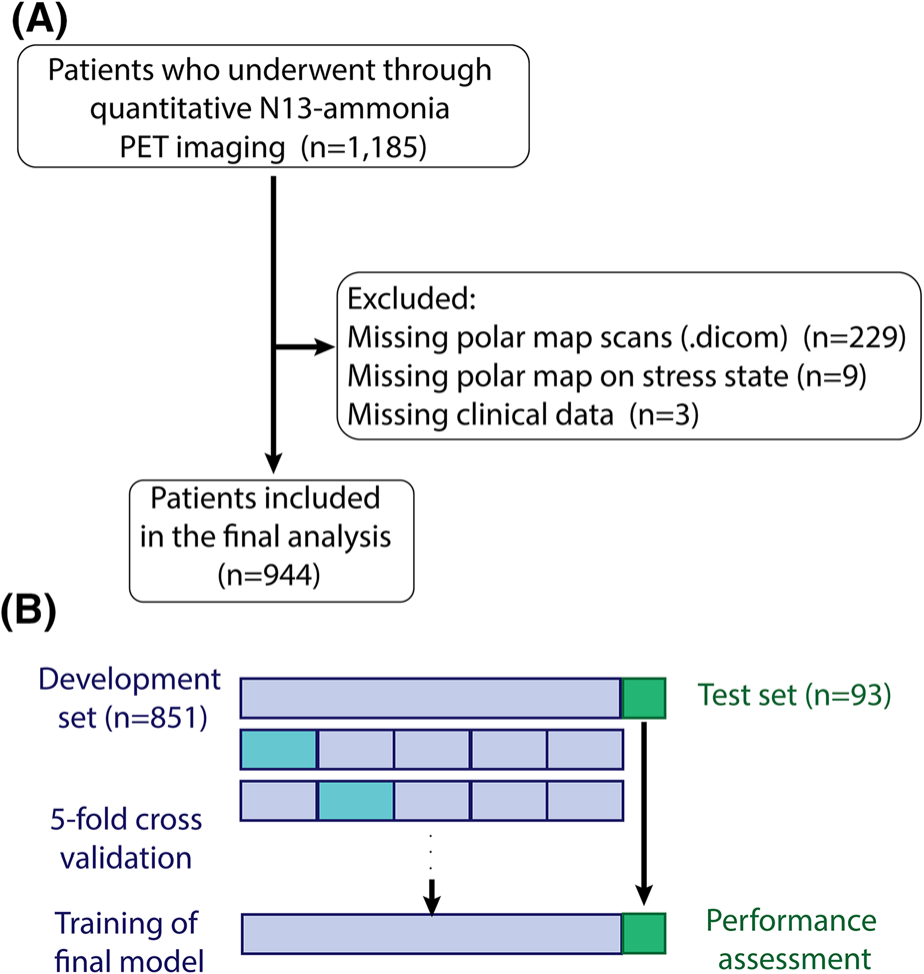
Study population and training strategy. (**A**) Study population. After quality control, 944 patients were included in the analysis. (**B**) Training strategy. 944 patients were randomly split into development set and test set in − 9:1 ratio. A 5-fold cross-validation was performed to optimize the hyperparameters; the final models were then trained on the whole development set and their performance were assessed using the unseen test set

Data were randomly divided into a *development* (training and validation) set and a *test* set which consisted of 90% and 10% of the total sample, respectively. Training and validation of the deep learning (DL) models were performed on the *development* set and a 5-fold cross-validation was employed to tune the hyperparameters of the DL models. The optimized models were evaluated on the *test* set, with data from individuals that had not been seen by the model during the training and validation process. Figure 1 depicts the implemented workflow.

The quantitative myocardial perfusion polar maps, namely the *rest*, *stress* and *reserve* polar maps derived from the PET scan were extracted in RGB color code (228 × 228 pixels wide) and resized to 224 × 224 pixels wide, which corresponds to the expected input dimension of the pretrained DL models. Separately, we developed classification models either from individual polar maps or the stack of all three (*rest*, *stress* and *reserve*) by concatenation.

#### Deep learning model architecture

We employed a modified ResNet-50 architecture and input the perfusion polar maps of each patient to predict mean segmental myocardial perfusion and identify (predict) in separate models an impaired MFR (<2.0) and the binary cardiovascular risk factors sex, positive smoking status, hypertension, dyslipidemia and diabetes mellitus through fine-tuning (*see Multi-task learning below*).

**Figure 2:**
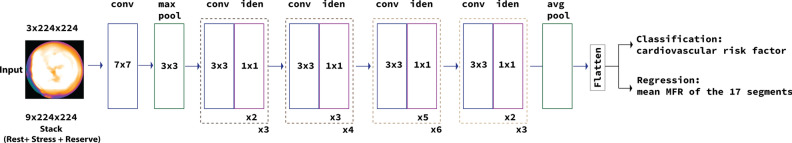
Modified ResNet-50 architecture with multi-task learning. The ResNet-50 was modified at the output layer for joint learning of classification task and regression task. The ratio of classification loss and regression loss as an additional hyperparameter was optimized in the cross-validation phase. For model using stack of three polar maps, the input layer was adjusted correspondingly (9 × 224 × 224). The main layers are represented in rectangles with solid lines, with numbers within describing their shapes and numbers outside indicate number of repeats respectively; the main stages are represented in rectangles with dotted lines. Avg pool: average pooling; conv: convolution block; iden: identity block; max pool: max pooling; MFR: myocardial flow reserve

Briefly, DL ResNet models are feedforward convolutional neural networks with “shortcut connections” between earlier layers and layers further down the network, called skip connections. ResNet models are organized into groups of layers, surrounded by the beginning and ending of a skip connection, called residual blocks, and variants of ResNet models are created by varying the number of such blocks (Figure 2). Thus, in the current study, we modified the last layer of the 50-layers ResNet-50 network to generate 19 output features, of which 17 were used to predict the mean MFR and the remaining two features for the aforementioned binary classifications. In the case of stacked polar maps, the input layer of models was modified accordingly.

#### Multi-task learning

To restrict the learning context for improved generalization, multi-task learning was employed such that each model learned to regress the mean MFR, while simultaneously identify an impaired MFR or individual cardiovascular risk factors (traits) (Figure 2). More specifically, the regression tasks of mean MFR guided the DL models to recognize the polar map in the context of the standardized 17-segmentation model; the models were to learn to master the classification task conditioned on the 17-segmentation model. Cross-entropy loss was selected for the classification task and mean squared error loss was selected for the regression task. The total was a weighted sum of the two losses, while λ ~ [0,1] was the hyperparameter to be optimized in cross-validation.

#### Transfer learning

A two-step transfer learning strategy was applied as follows: Model with parameters pre-trained on the ImageNet dataset was first finetuned to recognize the characteristics of polar maps via identification of impaired regional MFR and further tuned to classify individual cardiovascular risk factors.

The model parameters were optimized through back-propagation, using a variant of the adaptive stochastic gradient based optimization algorithm Adam,^11^ with a decoupled weight decay regularization.^12^ Considering the large number of parameters of ResNet-50 and the relatively small size of development dataset, we optimized only parameters of the last 3 layers of ResNet-50 for binary classifications. To further avoid overfitting of the model to training data, we applied data augmentation techniques, including limited rescaling (10%), rotation (±10°) and random dropout of pixels. All DL experiments were implemented on PyTorch 1.4.0.^13^

#### Attention heat maps

To explore and discuss patterns corresponding to the inherent relationships between the polar maps and cardiovascular risk factors identified by DL, we generated attention heat maps for each risk factor taking individuals from the test set. Given a predicted label (presence or absence of a specific risk factor), an attention heat map visualizes the relative importance (attribution) of pixels of the input image towards that label predicted by the DL model. We applied two different attribution approaches to generate the attention heatmap: a perturbation-based occlusion sensitivity method^14^ using a square patch of size 30×30 pixels and a gradient-based method GradCAM^15^ implemented using Captum,^16^ which is an open source python library for model interpretability. Briefly, in the perturbation-based method, the image is systematically occluded partially by sliding a black square along the image to examine how the model would (re-)classify. Areas that would change the classification with greater degree are then considered to be important. In the gradient-based method, the importance of input neurons (pixels) is assigned based on the gradient information flowing into the last convolutional layer of the neural network with respect to the target classification. Areas (pixel collections) with higher gradients are thus considered to be more important to the target classification. Attention maps based on high confidence predictions (>0.9 or the highest confidence in the absence of high confidence prediction) were visually evaluated by a clinician to search for potentially interpretable and spatially relevant patterns.

### Statistical Analysis

Descriptive statistics were expressed as frequency (percentage) for categorical variables, mean ± standard deviation (SD) for normally distributed quantitative variables and median (interquartile range, IQR) for variables with non-normal distributions. The normality of continuous variables was assessed by skewness statistics and graphically by histograms. Independent t-tests were used for continuous variables, while Pearson chi-squared tests were used for categorical variables to compare the differences between the patients with/without impaired MFR, and between the development and test set respectively. Statistical analyses were performed using Stata 16 (StataCorp LLC). A two-tailed p <0.05 was considered to be statistically significant.

#### Performance Evaluation of DL Model

Performance of the DL models was assessed by accuracy and area under the receiver operating curve (AUC) in the hold-out test set of 93 patients. A random prediction corresponded to an accuracy of 50%, and an AUC of 0.5 respectively. The 95% confidence intervals of both metrics were estimated by bootstrapping 4000 times. To compare performance to conventional statistical methods, logistic regression models for the cardiovascular risk factors were fitted with the mean MBF (rest and stress polar map) or MFR (reserve polar map) of the 17 segments using the training set. Thereon, DL models were contrasted against these regressions in the hold-out test set.

## Results

### Study Population Characteristics

A total of 944 patients were included in the analysis. Table 1 shows the clinical characteristics of the cohort, stratified by an impaired MFR (<2.0) as determined by the PET scan. Mean age was 65.3±9.2 in patients with no MFR impairment and 68.6±9.5 in patients with an impaired MFR. Significantly more men than women demonstrated an impaired MFR (no impaired MFR vs impaired MFR: 54.4% vs 46.2%, *P*-value=0.019). Patients with an impaired MFR were more likely to have diabetes (no impaired MFR vs impaired MFR: 10.8% vs 17.6%, *P* value = 0.007) and hypertension (no impaired MFR vs impaired MFR: 55.0% vs 46.3%, *P*-value=0.011), while no statistically significant differences in smoking behavior and dyslipidemia were observed. The cohort was randomly assigned to either the *development* (i.e., training and validation) or the *test* dataset in a 9:1 proportion, respectively (Figure 1). Table 2 presents the prevalence of cardiovascular risk traits (factors) in the *development* set and *test* sets, which proved comparable as expected from the random parcellation.

**Table 1:** Clinical characteristics of the study population

Characteristics	No impaired MFR	Impaired MFR	*P*-value
*N*	307	637	
Age, mean (SD)	65.3 (9.2)	68.6 (9.5)	**<0.001**
Sex (Female)	167 (54.4%)	294 (46.2%)	**0.019**
BMI (kg/m^2^), mean (SD)	27.2 (4.6)	27.7 (4.8)	0.087
Family history of coronary artery disease	81 (26.4%)	182 (28.6%)	0.480
Smoker	50 (16.3%)	78 (12.3%)	0.093
Diabetes	33 (10.8%)	112 (17.6%)	**0.007**
Dyslipidaemia	97 (31.6%)	216 (33.9%)	0.480
Hypertension	142 (46.3%)	350 (55.0%)	**0.011**
Duke score, median (IQR)	49 (22, 74)	54 (22, 77)	0.180
Rest LVEF, median (IQR)	68 (63, 74)	69 (61, 75)	0.640
Stress LVEF, median (IQR)	70 (64, 75)	70 (61, 75)	0.320

Significant differences between groups with *p* < 0.05 are indicated in bold

Impaired MFR: < 2 ml/g/min in at least one of the 17 segments from the American Heart Association/American College of Cardiology standardized myocardial segmentation model

*BMI* body mass index; *IQR* interquartile range; *LVEF* left ventricular ejection fraction; *SD* standard deviation

**Table 2:** Prevalence of clinical risk factors in development set and test set

Characteristics	Development set	Test set	*P* value
*N*	851	93	
Sex (female)	417 (49.1%)	44 (47.3%)	0.75
Smoker	114 (13.4%)	14 (15.1%)	0.66
Diabetes	130 (15.3%)	15 (16.1%)	0.84
Dyslipidaemia	286 (33.6%)	27 (29.0%)	0.37
Hypertension	449 (52.8%)	43 (46.2%)	0.23

### DL in Identifying an Impaired MFR

Table 3 shows the performance of the DL in detecting an abnormal myocardial perfusion, in either one of three territories or any of the territories. The highest performance was achieved among DL models either considering single reserve polar maps or the three polar maps stacks (*rest*, *stress* and *reserve*) as input, while the lowest performance was observed in those using rest polar maps as input. There was no significant difference in performance with regard to location of abnormal perfusion, either on specific a territory or overall. The DL model using myocardial perfusion reserve polar maps had the highest accuracy of 92.5% (95% confidence interval, CI 87.1-93.5%) in identifying an abnormal perfusion with an AUC = 0.94 [0.87, 0.98]. In contrast, the lowest DL accuracy was observed in the model using the polar maps from only the rest state to detect abnormality in the LAD territory (accuracy of 54.8% [44.1, 64.5], AUC = 0.54 [0.43, 0.64]) (Table 3) as expected.

**Table 3:** Performance of DL models on identification of impaired MFR

Output	Prevalence (%)	Accuracy (%) using Rest PM	AUC using Rest PM	Accuracy (%) using Stress PM	AUC using Stress PM	Accuracy (%) using Reserve PM	AUC using Reserve PM	Accuracy (%) using stacked PMs	AUC using stacked PMs
Impaired MFR in any segment	67.50	**63.4**	**0.68**	**72**	**0.68**	**92.5**	**0.94**	**89.2**	**0.90**
		**(52.7–73.1)**	**(0.57–0.78)**	**(62.4–80.6)**	**(0.55–0.79)**	**(87.1–97.8)**	**(0.87–0.98)**	**(82.8–94.6)**	**(0.82–0.96)**
Impaired MFR in LAD	52.50	54.8	0.54	**75.3**	**0.76**	**87.1**	**0.89**	**88.2**	**0.90**
		(44.1**–**64.5)	(0.43**–**0.64)	**(66.7–83.9)**	**(0.66–0.85)**	**(79.6–93.5)**	**(0.83–0.94)**	**(81.7–94.6)**	**(0.84–0.95)**
Impaired MFR in RCA	57.40	58.1	**0.61**	**72**	**0.73**	**88.2**	**0.87**	**91.4**	**0.91**
		(48.4**–**67.7)	**(0.52–0.71)**	**(63.4–80.6)**	**(0.64–0.82)**	**(81.7–94.6)**	**(0.80–0.94)**	**(84.9–96.8)**	**(0.84–0.97)**
Impaired MFR in LCx	36.70	**63.4**	**0.63**	**77.4**	**0.70**	**92.5**	**0.92**	**91.4**	**0.92**
		**(53.8–73.1)**	**(0.52–0.73)**	**(68.8–86.0)**	**(0.62–0.79)**	**(87.1–97.8)**	**(0.86–0.98)**	**(84.9–96.8)**	**(0.86–0.97)**

Performance with 95% confidence interval not covering the expected performance of a random prediction model are indicated in bold

Impaired MFR was defined as MFR< 2.0 ml/g/min

*AUC* area under curve; *DL* deep learning; *LAD* left anterior descending artery; *LCx* left circumflex artery; *MFR* myocardial flow reserve; *PM* polar map; *RCA* right coronary artery

### DL in Cardiovascular Risk Trait Classification

Thereon, DL models were further finetuned to identify the presence or absence of the specified cardiovascular risk traits (factors). Identification of sex was notably successful regardless of the input (single or stacked polar maps) with the highest performance observed in rest polar maps: accuracy = 80.6% [72.0, 88.2] and an AUC = 0.81 [0.73, 0.88]. Notably, the DL model analyzing rest polar maps achieved an accuracy of 86.0% (95% CI 78.5-92.5) and an AUC of 0.71 (95% CI 0.58-0.85) in identifying a positive smoking status. DL models for detection of diabetes performed only marginally better than random with the highest performance found in the model using polar maps from reserve state with an accuracy = 77.4% [68.8-86.0%] and AUC = 0.65 [0.51, 0.79]. The identification of dyslipidemia and arterial hypertension showed the lowest performance and was not statistically significant. The expanded results are shown in Table 4.

**Table 4:** Performance of DL models on identification of cardiovascular risk factors

Output	Prevalence (%)	Accuracy (%) using Rest PM	AUC using Rest PM	Accuracy (%) using Stress PM	AUC using Stress PM	Accuracy (%) using Reserve PM	AUC using Reserve PM	Accuracy (%) using stacked PMs	AUC using stacked PMs
Diabetes	15.4	**80.6**	0.62	**80.6**	0.62	**77.4**	**0.65**	54.8	0.52
		**(72.0–88.2)**	(0.50**–**0.75)	**(72.0–88.2**)	(0.49**–**0.75)	**(68.8–86.0)**	**(0.51–0.79)**	(44.1**–**64.5)	(0.38**–**0.66)
Dyslipidaemia	33.2	**65.6**	0.52	59.1	0.46	52.7	0.54	55.9	0.58
		**(55.9–75.3)**	(0.44**–**0.60)	(49.5**–**68.8)	(0.38**–**0.55)	(43.0**–**62.4)	(0.42**–**0.64)	(45.2**–**65.6)	(0.47**–**0.69)
Arterial hypertension	52.2	47.3	0.47	**60.2**	0.60	**60.2**	0.60	**60.2**	0.59
		(37.6**–**57.0)	(0.37**–**0.58)	**(50.5–69.9)**	(0.50**–**0.69)	**(50.5–69.9)**	(0.49**–**0.69)	**(50.5–69.9)**	(0.50**–**0.69)
Sex	48.9 female	**80.6**	**0.81**	**65.6**	**0.65**	**65.6**	**0.65**	**66.7**	**0.66**
		**(72.0–88.2)**	**(0.73–0.88)**	**(55.9–75.3)**	**(0.55–0.75)**	**(54.8–75.3)**	**(0.55–0.74)**	**(57.0–76.3)**	**(0.57–0.75)**
Smoking	13.6	**86.0**	**0.71**	**75.3**	0.47	**77.4**	0.49	**74.2**	0.50
		**(78.5–92.5)**	**(0.58–0.85)**	**(66.7–83.9)**	(0.41**–**0.56)	**(68.8–86.0)**	(0.42**–**0.58)	**(64.5–82.8)**	(0.41**–**0.61)

Performance with 95% confidence interval not covering the expected performance of a random prediction model are indicated in bold

*AUC* area under curve; *DL* deep learning; *PM* polar map

When compared against classical regression models, DL models attained similar performance in identification of sex and diabetes with the exception the DL model also able to identify sex using reserve polar maps as input (Table 5). Notably, classical regression models were not able to identify positive smoking status taking mean MFR as input.

**Table 5:** Performance of logistic regression models on identification of impaired MFR and cardiovascular risk factors

Output	Prevalence (%)	AUC using rest PM	AUC using stress PM	Reserve AUC using reserve PM	AUC using stacked PMs
Impaired MFR in any segment	67.50	**0.70**	**0.82**	**0.94**	**0.98**
		**(0.60–0.81)**	**(0.73–0.90)**	**(0.90–0.98)**	**(0.96–1.00)**
Diabetes	15.40	0.6	0.5	**0.61**	0.58
		(0.44**–**0.76)	(0.32**–**0.68)	**(0.45–0.78)**	(0.41**–**0.75)
Dyslipidaemia	33.20	0.55	0.45	0.44	0.5
		(0.41**–**0.69)	(0.32**–**0.58)	(0.31**–**0.57)	(0.38**–**0.63)
Arterial hypertension	52.20	0.52	0.57	0.54	0.53
		(0.40**–**0.64)	(0.45**–**0.68)	(0.42**–**0.66)	(0.41**–**0.65)
Sex female	48.90	**0.85**	**0.65**	0.57	**0.79**
		**(0.77–0.93)**	**(0.54–0.76)**	(0.45**–**0.69)	**(0.69–0.88)**
Smoking	13.60	0.44	0.43	0.56	0.48
		(0.29**–**0.60)	(0.27**–**0.60)	(0.39**–**0.73)	(0.31**–**0.65)

Performance with 95% confidence interval not covering the expected performance of a random prediction model are indicated in bold

*AUC* area under curve; *PM* polar map

### DL Attention Maps Evaluation

**Figure 3:**
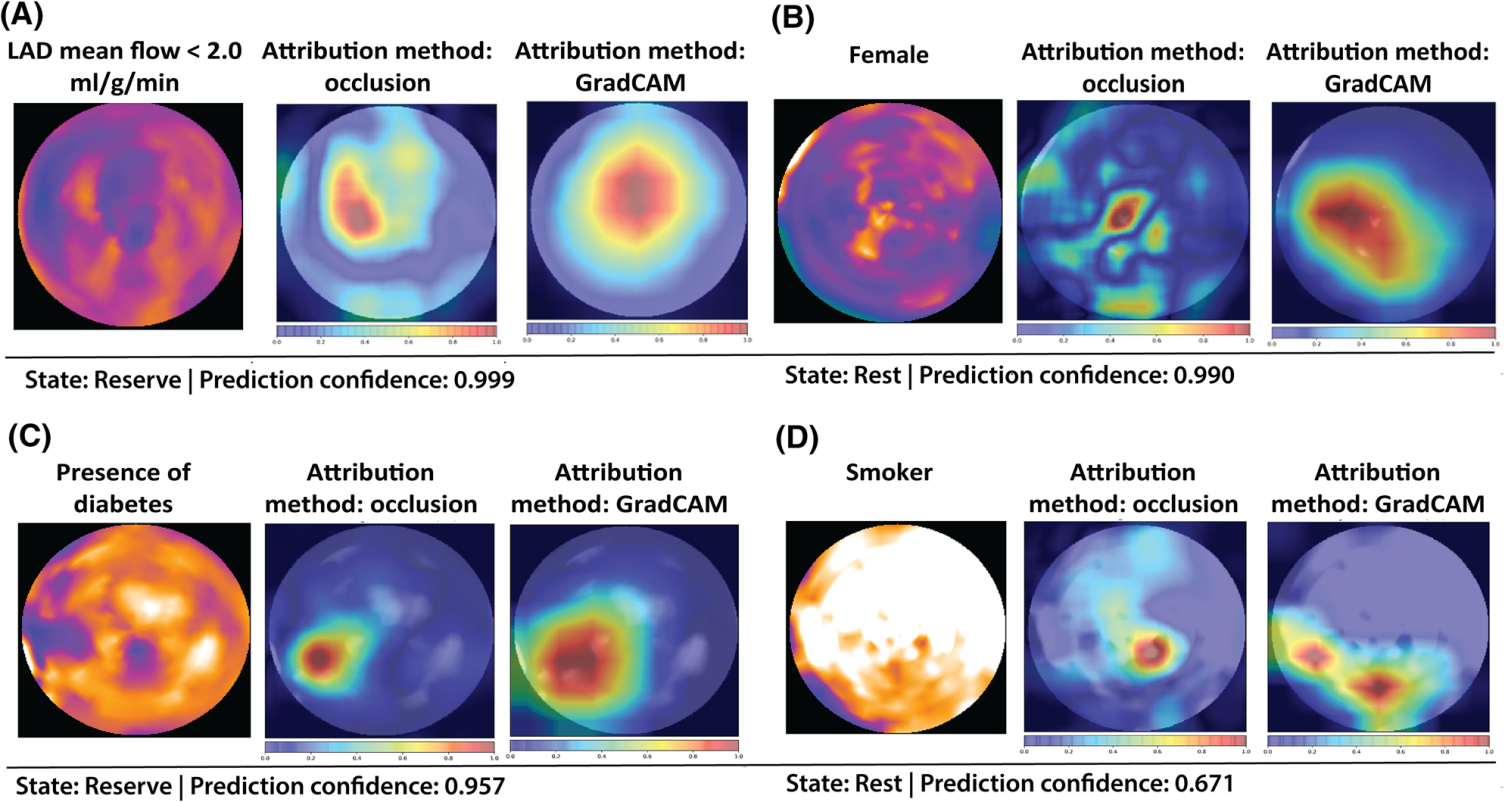
Attention heatmaps. (**A**) Attention heatmap for model prediction of impaired regional mean MFR in left anterior descending artery (LAD). (**B**) Attention heatmap for model prediction of female. (**C**) Attention heatmap for model prediction of diabetes. (**D**) Attention heatmap for model prediction of smoking. Each attention map was generated with one representative polar map from a patient from the test set previously unseen by the model in the development process

To explore the localizability and spatial profile of the associations captured by DL for the identification of cardiovascular risk traits, attention heatmaps were generated from the top performing statistically significant models, namely those classifying sex, diabetes mellitus and smoking status. The attention maps placed on the polar maps with the highest prediction confidence showed that female sex identification hovered over the apical regions of the left ventricle (Figure 3). Conversely, we observed no fixed regions highlighted for the identification of diabetes mellitus and smoking for which rather diffuse patterns were noted.

## Discussion

The present study documents the feasibility and performance of a multi-task DL approach in the evaluation of quantitative PET myocardial perfusion polar maps for the identification of an impaired MFR and the identification of common cardiovascular risk traits (factors) in subjects with known or suspected CAD at the individual patient-level. Furthermore, our results frame how DL may enhance our capacity to identify complex attributes that associate with known risk factors that affect myocardial perfusion beyond what conventional regression analysis utilizing myocardial blood flow estimations may offer.

The clinical value of cardiac functional imaging is undisputed. PET allows quantitative evaluation of myocardial perfusion in absolute terms for the characterization of ischemia in CAD. Furthermore, perfusion estimates are also influenced by well-known cardiovascular risk factors, namely sex, smoking, dyslipidemia, arterial hypertension and diabetes mellitus. These traits are understood to additively modify risk at the individual patient level as underlined by the concept of clinical likelihood in the latest European Society of Cardiology guidelines on the diagnosis and management of chronic coronary syndromes.^17^ The diagnostic and prognostic value of myocardial perfusion quantification beyond that of robust factors such as LVEF, scar extent, and even semi-quantitative perfusion variables, such as the summed stress score, has been illustrated through traditional statistical analyses. In fact, quantitative myocardial perfusion estimates (namely, stress MBF and MFR) have been suggested to represent two of the most significant predictors of cardiac events.^18^ In this study, DL showed the best performance to accurately identify abnormal myocardial perfusion through the evaluation of *reserve* polar maps both regionally and globally. This is relevant because it will allow us to incorporate its utility into decision support for the clinical evaluation of PET myocardial perfusion scans.

On the other hand, there is paucity in previous studies reporting sex differences in global MBF values, and differences in the resulting MFR value have been inconclusive.^19,20^ In the current study, we found that it was possible to classify the sex of a patient from either rest, stress or reserve polar maps, where DL achieved the best performance when by only evaluating single rest polar maps. Attention heatmaps showed apical regions of the left ventricle to be an area of interest in such distinction. This result suggests that there may be intrinsic differences between males and females leading to divergent perfusion patterns during rest. Furthermore, we found that the DL model showed a discriminatory performance (AUC > 0.5) in identifying a positive smoking status and diabetes mellitus all at an individual level. However, this was not the case for the classification of arterial hypertension and dyslipidemia. Whether this was a result of differences in the average profile of adjacent cardiovascular risk factors remains unclear and should be cautiously considered. This differential performance may also arise from the fact that the effects of hypertension and dyslipidemia on myocardial perfusion will also be dependent on their degree of severity and on whether these conditions are being medically treated. Unfortunately, such information was not directly available in this study. Yet, we believe that such factors may have moderated the association of the risk traits with MBF and MFR, and thus affected the classification capacity of DL. This suggestion aligns with the fact that strongest differentiation could be made in the identification of sex already discussed.

It must be understood that the conventional approach of operationalizing information provided by myocardial perfusion imaging (e.g. PET) into simplified categorical (e.g. the semi-quantitative 5-point scale) or absolute continuous variables (e.g. MFR in ml/g/min) merely represents a heuristic that facilitates human interpretation and application of linear statistics. Furthermore, images in any domain represent by themselves a very complex collection of patterns emerging from all relationships between their smallest addressable elements, i.e. pixels. It is likely, therefore, that relevant features within comprehensive perfusion images may be overlooked by such operationalization.

Overall, this DL study offers a novel way in which the intrinsic value of advanced cardiac imaging can be more extensively utilized for clinical (identification of ischemia and cardiovascular risk traits) and research (exploration of complex patterns in the classification of such factors) purposes. We recognize, however, that whether this can in fact improve risk stratification and event prediction remains to be elucidated.

DL is an advanced machine learning methodology, able to appraise and identify complex image patterns that may go undetected by the human eye. Our DL implementation adds to the evidence suggesting that high-quality myocardial perfusion images contain a substantial amount of information with value beyond that of their numerical summary extracts, and that these relate at least moderately with conventional cardiovascular risk factors that represent in themselves chronic co-morbidities. Although a precise description of such abstract patterns was not yet identified, further research to identify the interactions of these patterns and quantify their importance in the classification task is warranted.

The present study naturally carries all the intrinsic disadvantages of any observational study. It also deals with a complex DL algorithm for which interpretation can be considered more challenging to perform than simpler statistical methods. This can be an obstacle when clinical interpretation of intermediate features is needed. In the current study, we investigated whether information on cardiovascular risk traits could be inferred from PET polar maps through DL. To mitigate the issue of a relatively small sample size in the context of DL, we employed multi-task learning to guide the network towards relations connected to the flow patterns by training the models to predict (an impaired) MFR from the polar maps and then the risk factors. This served not only as a prior knowledge of the polar map to aid the learning process, but also forced the models to extract common features relevant to all tasks, therefore potentially enhancing clinical/biological meaning of the prediction result. As DL modelling substantially exceeds threshold rule-based classification in complexity (sheer number of input and parameters/coefficients) a perfect performance (AUC = 1.0) in the identification of an impaired MPR could not be achieved at this sample size. Nevertheless, the achieved performance may still be considered as good for the identification of myocardial ischemia while simultaneously contributing to the further classification of cardiovascular risk traits from the polar maps.

## New Knowledge Gained

Deep learning can be applied on quantitative PET myocardial perfusion polar maps to identify ischemia and extract information on cardiovascular risk factors namely, sex, smoking and diabetes. A priori knowledge can be injected to assist the training of a deep learning model.

## Conclusions

Multi-task DL for the evaluation of quantitative PET myocardial perfusion polar maps is able to identify an impaired MFR as well as cardiovascular risk traits at the individual-patient level. DL seems able to significantly identify sex, smoking and probably diabetes mellitus from both localized and diffuse perfusion patterns throughout the left ventricle. Although the mechanistic significance and clinical relevance of such patterns and identification capacity through DL analysis is still unclear, further research into the exploration of advanced cardiac imaging through DL is warranted.

### Disclosures and Funding

The work of M.W. Yeung and J.W. Benjamins was supported by the Research Project CVON-AI (2018B017), financed by the PPP Allowance made available by Top Sector Life Sciences & Health to the Dutch Heart Foundation to stimulate public-private partnerships.

## Supplementary Information

Below is the link to the electronic supplementary material.

Supplementary file1 (PPTX 7010 kb)
