# Supplementary material for: Multi-task Deep Learning of Myocardial Blood Flow and Cardiovascular Risk Traits from PET Myocardial Perfusion Imaging
Source: J Nucl Cardiol. 2022 Mar 10;29(6):3300–10. doi: 10.1007/s12350-022-02920-x (PMC9834343; doi:10.1007/s12350-022-02920-x)
Supplement: Supplementary file 1 — Supplementary file1 (PPTX 7010 kb) [file 12350_2022_2920_MOESM1_ESM.pptx]

## Slide 1
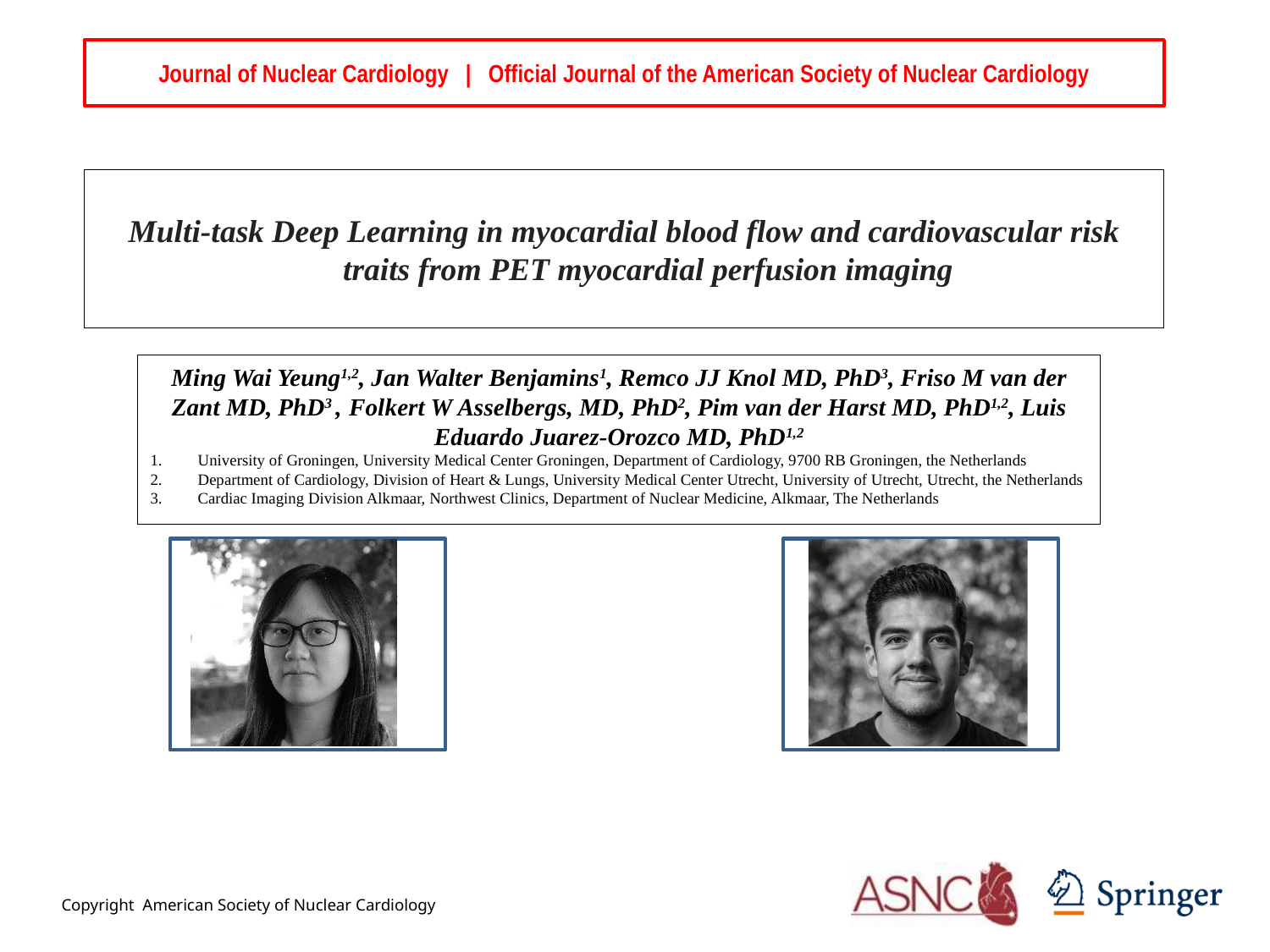

Journal of Nuclear Cardiology | Official Journal of the American Society of Nuclear Cardiology
# Multi-task Deep Learning in myocardial blood flow and cardiovascular risk traits from PET myocardial perfusion imaging
Ming Wai Yeung1,2, Jan Walter Benjamins1, Remco JJ Knol MD, PhD3, Friso M van der Zant MD, PhD3 , Folkert W Asselbergs, MD, PhD2, Pim van der Harst MD, PhD1,2, Luis Eduardo Juarez-Orozco MD, PhD1,2
University of Groningen, University Medical Center Groningen, Department of Cardiology, 9700 RB Groningen, the Netherlands
Department of Cardiology, Division of Heart & Lungs, University Medical Center Utrecht, University of Utrecht, Utrecht, the Netherlands
Cardiac Imaging Division Alkmaar, Northwest Clinics, Department of Nuclear Medicine, Alkmaar, The Netherlands
Head shot of author
required
Institution
Picture/Logo
Optional
Copyright American Society of Nuclear Cardiology

## Slide 2
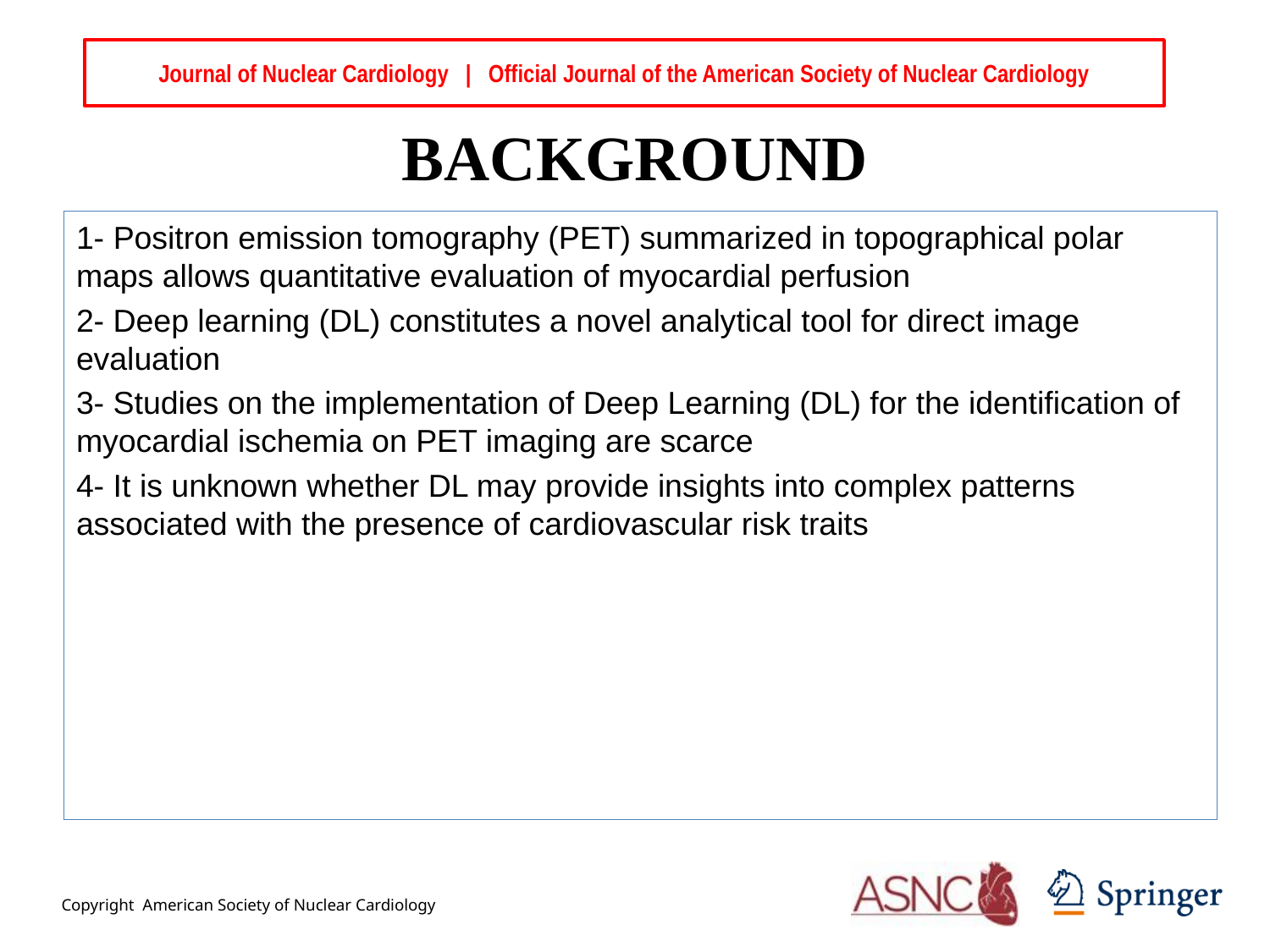

Journal of Nuclear Cardiology | Official Journal of the American Society of Nuclear Cardiology
# BACKGROUND
1- Positron emission tomography (PET) summarized in topographical polar maps allows quantitative evaluation of myocardial perfusion
2- Deep learning (DL) constitutes a novel analytical tool for direct image evaluation
3- Studies on the implementation of Deep Learning (DL) for the identification of myocardial ischemia on PET imaging are scarce
4- It is unknown whether DL may provide insights into complex patterns associated with the presence of cardiovascular risk traits
Copyright American Society of Nuclear Cardiology

## Slide 3
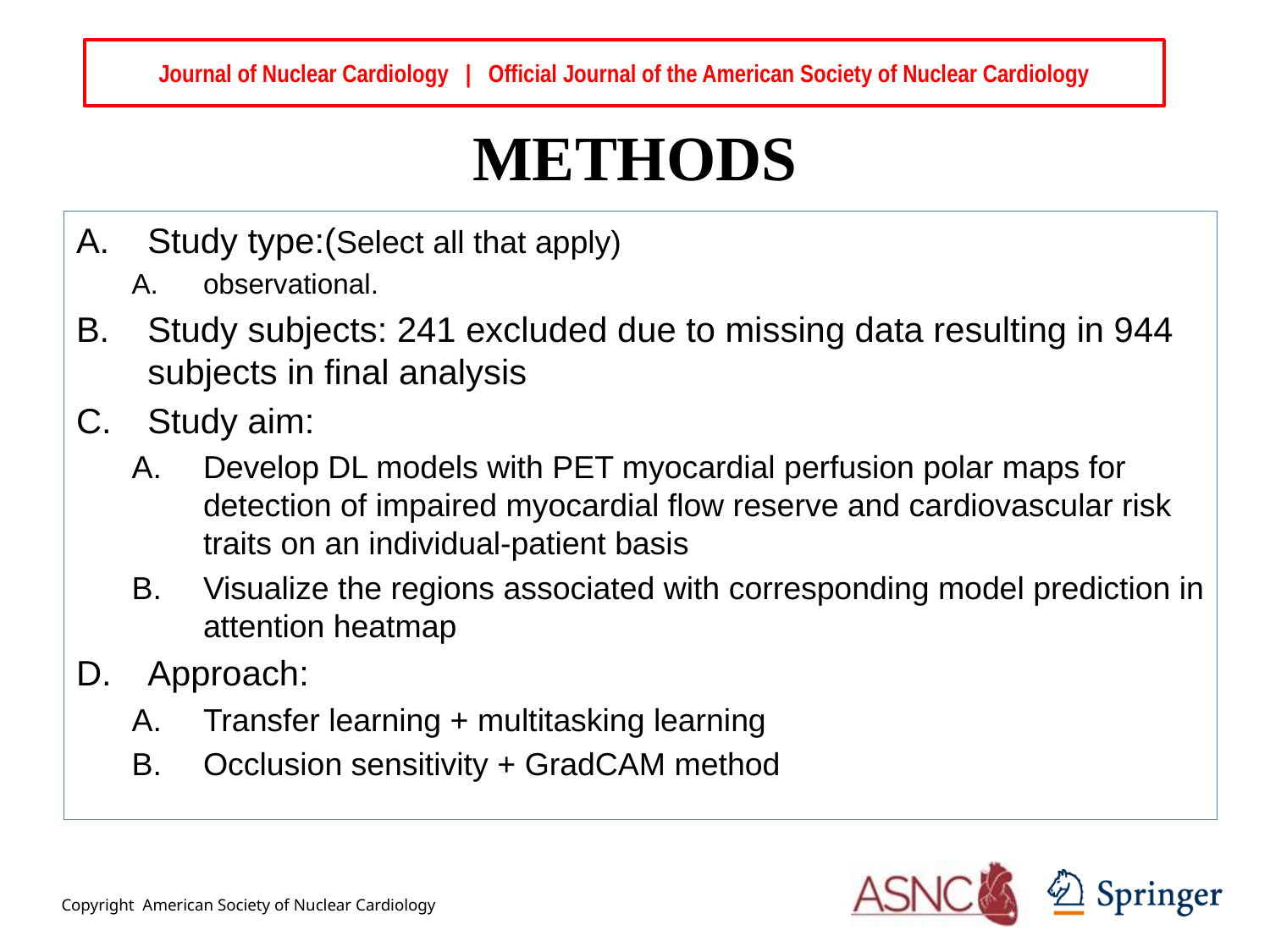

Journal of Nuclear Cardiology | Official Journal of the American Society of Nuclear Cardiology
# METHODS
Study type:(Select all that apply)
observational.
Study subjects: 241 excluded due to missing data resulting in 944 subjects in final analysis
Study aim:
Develop DL models with PET myocardial perfusion polar maps for detection of impaired myocardial flow reserve and cardiovascular risk traits on an individual-patient basis
Visualize the regions associated with corresponding model prediction in attention heatmap
Approach:
Transfer learning + multitasking learning
Occlusion sensitivity + GradCAM method
Copyright American Society of Nuclear Cardiology

## Slide 4
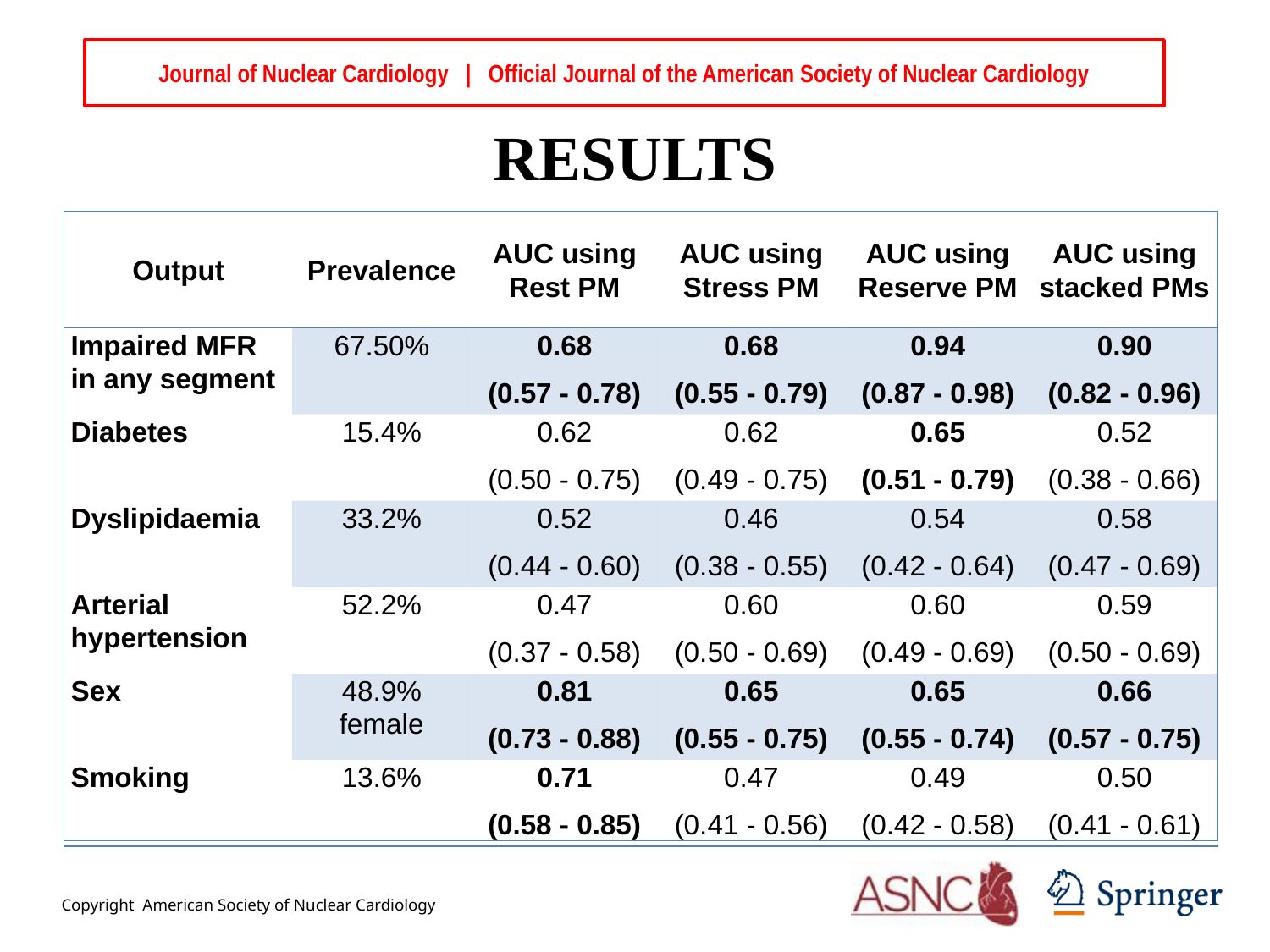

Journal of Nuclear Cardiology | Official Journal of the American Society of Nuclear Cardiology
# RESULTS
| Output | Prevalence | AUC using Rest PM | AUC using Stress PM | AUC using Reserve PM | AUC using stacked PMs |
| --- | --- | --- | --- | --- | --- |
| Impaired MFR in any segment | 67.50% | 0.68 (0.57 - 0.78) | 0.68 (0.55 - 0.79) | 0.94 (0.87 - 0.98) | 0.90 (0.82 - 0.96) |
| Diabetes | 15.4% | 0.62 (0.50 - 0.75) | 0.62 (0.49 - 0.75) | 0.65 (0.51 - 0.79) | 0.52 (0.38 - 0.66) |
| Dyslipidaemia | 33.2% | 0.52 (0.44 - 0.60) | 0.46 (0.38 - 0.55) | 0.54 (0.42 - 0.64) | 0.58 (0.47 - 0.69) |
| Arterial hypertension | 52.2% | 0.47 (0.37 - 0.58) | 0.60 (0.50 - 0.69) | 0.60 (0.49 - 0.69) | 0.59 (0.50 - 0.69) |
| Sex | 48.9% female | 0.81 (0.73 - 0.88) | 0.65 (0.55 - 0.75) | 0.65 (0.55 - 0.74) | 0.66 (0.57 - 0.75) |
| Smoking | 13.6% | 0.71 (0.58 - 0.85) | 0.47 (0.41 - 0.56) | 0.49 (0.42 - 0.58) | 0.50 (0.41 - 0.61) |
Copyright American Society of Nuclear Cardiology

## Slide 5
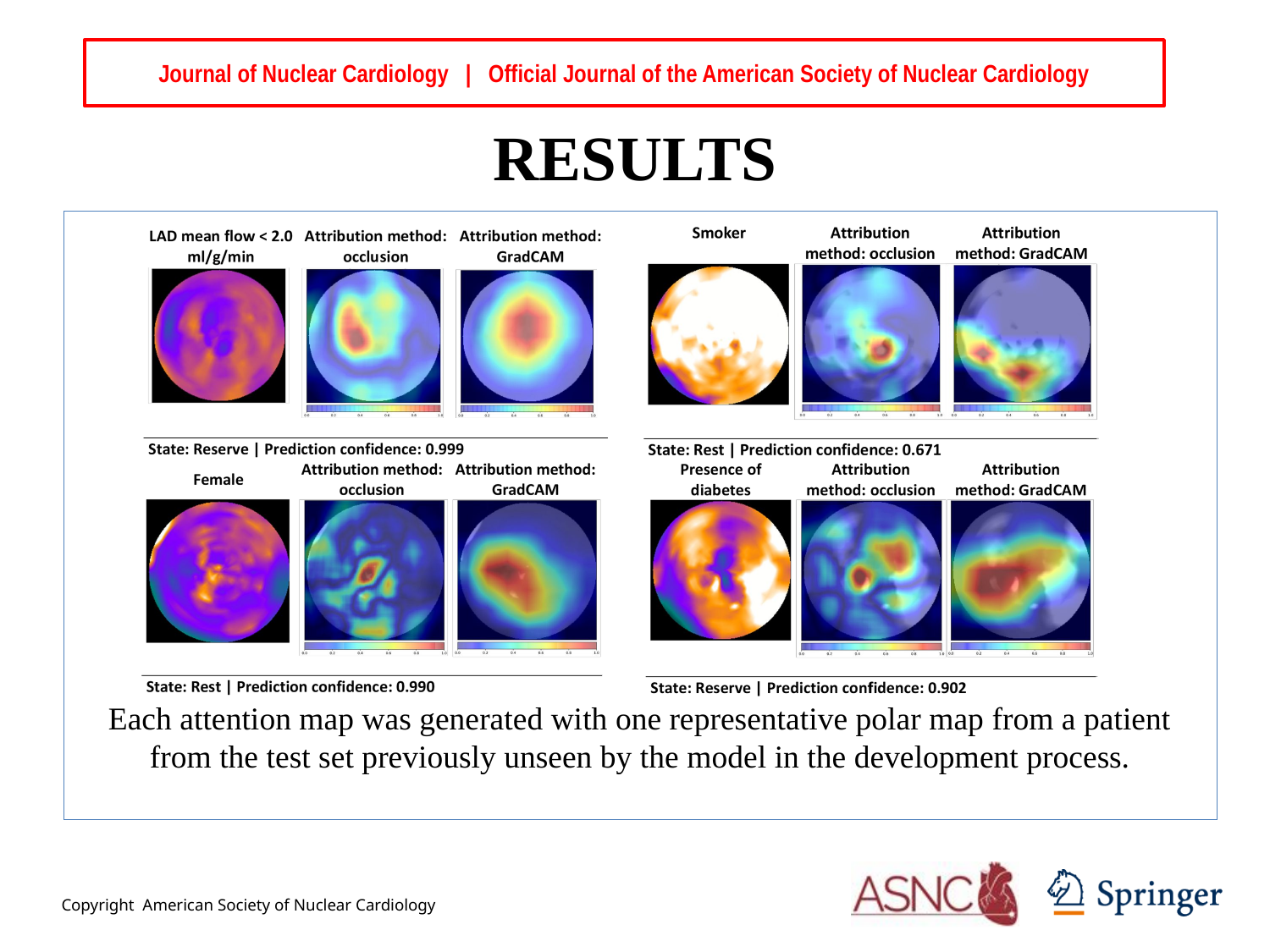

Journal of Nuclear Cardiology | Official Journal of the American Society of Nuclear Cardiology
# RESULTS
Each attention map was generated with one representative polar map from a patient from the test set previously unseen by the model in the development process.
Copyright American Society of Nuclear Cardiology

## Slide 6
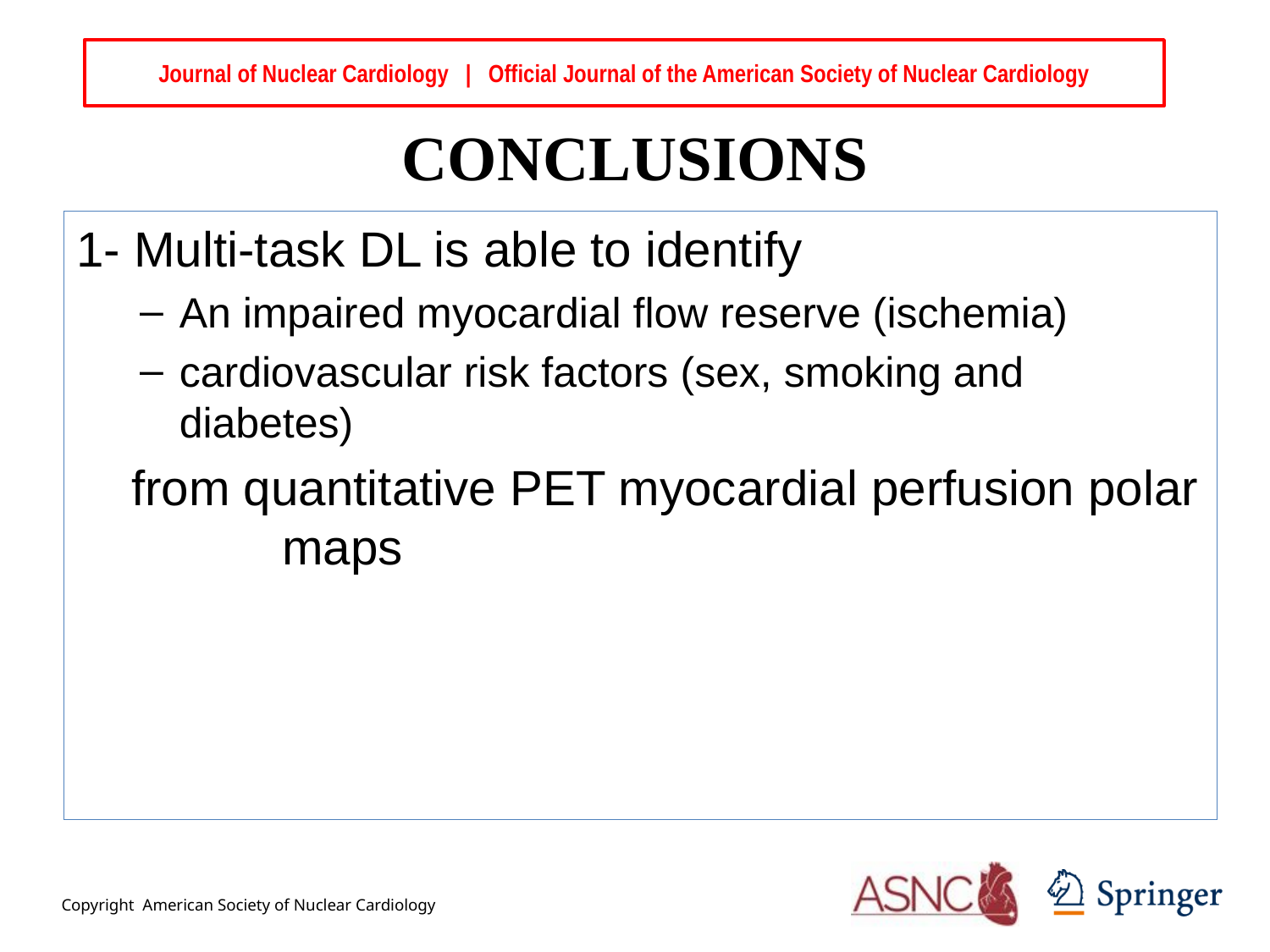

Journal of Nuclear Cardiology | Official Journal of the American Society of Nuclear Cardiology
# CONCLUSIONS
1- Multi-task DL is able to identify
An impaired myocardial flow reserve (ischemia)
cardiovascular risk factors (sex, smoking and diabetes)
 from quantitative PET myocardial perfusion polar maps
Copyright American Society of Nuclear Cardiology
